# Enriched environment attenuates hippocampal theta and gamma rhythms dysfunction in chronic cerebral hypoperfusion via improving imbalanced neural afferent levels

**DOI:** 10.3389/fncel.2023.985246

**Published:** 2023-05-17

**Authors:** Jiaxin Zheng, Sisi Peng, Lingling Cui, Xi Liu, Tian Li, Zhenyu Zhao, Yaqing Li, Yuan Hu, Miao Zhang, Linling Xu, JunJian Zhang

**Affiliations:** ^1^Department of Neurology, Zhongnan Hospital of Wuhan University, Wuhan, China; ^2^Department of Anesthesiology, Tongren Hospital of Wuhan University, Wuhan, China; ^3^Clinical Medical Research Center for Dementia and Cognitive Impairment in Hubei Province, Wuhan, China

**Keywords:** chronic cerebral hypoperfusion, enriched environment, cognitive dysfunction, neural oscillations, phase amplitude coupling, neurotransmitter balance

## Abstract

Chronic cerebral hypoperfusion (CCH) is increasingly recognized as a common cognitive impairment-causing mechanism. However, no clinically effective drugs to treat cognitive impairment due to CCH have been identified. An abnormal distribution of neural oscillations was found in the hippocampus of CCH rats. By releasing various neurotransmitters, distinct afferent fibers in the hippocampus influence neuronal oscillations in the hippocampus. Enriched environments (EE) are known to improve cognitive levels by modulating neurotransmitter homeostasis. Using EE as an intervention, we examined the levels of three classical neurotransmitters and the dynamics of neural oscillations in the hippocampus of the CCH rat model. The results showed that EE significantly improved the balance of three classical neurotransmitters (acetylcholine, glutamate, and GABA) in the hippocampus, enhanced the strength of theta and slow-gamma (SG) rhythms, and dramatically improved neural coupling across frequency bands in CCH rats. Furthermore, the expression of the three neurotransmitter vesicular transporters—vesicular acetylcholine transporters (VAChT) and vesicular GABA transporters (VGAT)—was significantly reduced in CCH rats, whereas the expression of vesicular glutamate transporter 1 (VGLUT1) was abnormally elevated. EE partially restored the expression of the three protein levels to maintain the balance of hippocampal afferent neurotransmitters. More importantly, causal mediation analysis showed EE increased the power of theta rhythm by increasing the level of VAChT and VGAT, which then enhanced the phase amplitude coupling of theta-SG and finally led to an improvement in the cognitive level of CCH. These findings shed light on the role of CCH in the disruption of hippocampal afferent neurotransmitter balance and neural oscillations. This study has implications for our knowledge of disease pathways.

## 1. Introduction

Dementia is a significant public health concern (Prince et al., 2019), defined as the chronic, acquired loss of two or more cognitive functions caused by brain illness or damage (Arvanitakis et al., 2019). Chronic cerebral hypoperfusion (CCH) is the pathophysiological underpinning of several dementia subtypes (Raz et al., 2016). CCH causes progressive brain damage such as white matter damage (Selvaraji et al., 2022), neuroendocrine disruption (Lin et al., 2022), energy metabolism (Ren et al., 2021), and neuroinflammation (Poh et al., 2022). Despite recent improvements in clinical and experimental neuroscience, there are few therapy options for cognitive impairment prevention, and the processes underlying CCH-related cognitive impairment are unclear.

There is strong evidence that CCH-related cognitive impairment is reflected in the degradation of spatial learning and memory (Ran et al., 2020; Kim et al., 2021). The hippocampal formation is the most vulnerable brain region to cerebral ischemia, and normal firing of neuronal clusters in the hippocampus is essential for spatial learning and memory (Wilson and McNaughton, 1993; Sugawara et al., 2002). Because the parietal dendrites of the pyramidal neurons in the hippocampus are parallelly oriented, synaptic currents in the same direction readily create synchronized firing activity in clusters of neurons. This rhythmic electrophysiological activity is known as neural oscillation (Buzsaki, 2002).

Theta and gamma oscillations are the two primary neuronal oscillations in the hippocampus that substantially influence memory and cognitive ability (Buzsaki, 2002; Csicsvari et al., 2003). Theta rhythm (4–12 Hz) occurs during active behavior and rapid eye movement sleep and is considered to help the brain acquire and learn new information (Colgin, 2013). Gamma rhythms (30–100 Hz), which are lower in amplitude than theta rhythms, are recorded in the hippocampus during a range of behaviors (Colgin, 2016). There is substantial evidence linking theta and gamma rhythms and their interactions in the hippocampus to neurological and psychiatric diseases (Uhlhaas and Singer, 2010; Zheng and Zhang, 2015; Zott et al., 2018; Yakubov et al., 2022). Abnormalities in the theta rhythm contribute to amnesia and mild cognitive impairment (Gonzalez et al., 2022; Wang et al., 2022). Gamma rhythm and theta-gamma phase amplitude coupling (PAC) are closely linked to Alzheimer’s disease, attention deficit, and associative memory impairment in old age (Karlsson et al., 2022; Liu et al., 2022; Mehak et al., 2022). But less attention has been paid to the weird things that happen with neural oscillations and how they affect CCH. Furthermore, the mechanism by which this anomalous alteration occurs in CCH is unclear.

Neural oscillations in the hippocampus are modulated by afferent nerve fibers to the hippocampus *via* releasing various neurotransmitters. Cholinergic, GABAergic, and glutamatergic afferent nerve fibers are the three primary hippocampal afferent nerve fibers. They release the appropriate neurotransmitters that trigger and regulate the electrical activity of the hippocampus (Gahwiler and Brown, 1985; Margrie et al., 1998; Chapman and Lacaille, 1999; Leao et al., 2012; Bao et al., 2017). In a variety of disease models, disruption of hippocampal afferent neurotransmission causes oscillatory rhythm impairment (Gisabella et al., 2012; Nakao et al., 2015; Magno et al., 2017; Takeuchi et al., 2021; Davila-Bouziguet et al., 2022). In the model of CCH, the neurotransmitter system appears to be disturbed (Qu et al., 2014; Long et al., 2015; Wang et al., 2020), but its relationship with neural oscillations has not been investigated.

Enriched environment (EE) is a well-known non-pharmacological intervention that improves cognitive function and has been shown to aid in neurotransmitter homeostasis regulation (He et al., 2010; Wang et al., 2016). In our previous study, EE improved cognitive impairment caused by CCH (Li et al., 2022). However, the manner in which EE interferes with cognition remains an open subject that requires further investigation.

Therefore, we utilized a modified permanent bilateral common carotid artery occlusion (2-VO) model to examine changes in hippocampal neural afferents and hippocampal neural oscillations in CCH rats. We also used association analysis and mediation analysis to determine if EE could improve cognitive function by improving abnormal neural oscillations and ameliorating abnormal hippocampal afferent neurotransmitter concentrations in CCH.

## 2. Materials and methods

### 2.1. Animals

Adult male Sprague-Dawley (SD) rats (weight: 220–240 g, SPF Beijing Biotechnology Co., LTD.) were used in this study. The number of rats per cage was three. All experiments were carried out in accordance with the National Research Council’s Guide for the Care and Use of Laboratory Animals (Guide, NRC 2011) and the European Convention for the Protection of Vertebrate Animals Used for Experimental and Other Scientific Purposes (ETS 123), with the approval of the Wuhan University Center for Animal Experiment’s IACUC (IACUC Number: SQ20200054).

### 2.2. Design of experiment

Rats were randomly allocated, and the researchers were blinded to the grouping until the experiments were completed. Animals were randomly assigned to the following three experimental groups: (1) subjected to a sham procedure (Sham group); (2) subjected to a modified 2-VO surgery (CCH group); (3) subjected to a modified 2-VO surgery and EE (CCH+EE group).

**FIGURE 1:**
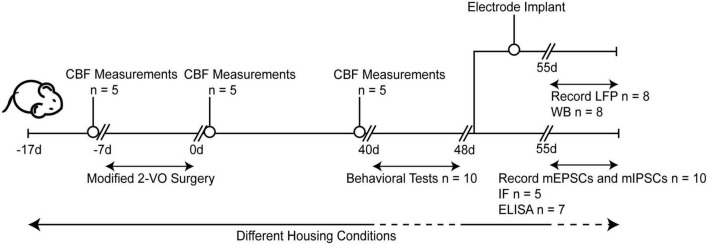
Experimental arrangement of this study. CBF, cerebral blood flow; 2-VO, permanent bilateral common carotid artery occlusion; d, day; LFP, local field potentials; WB, western blot; IF, immunofluorescence; ELISA, enzyme-linked immunosorbent assay; mEPSCs, miniature excitatory postsynaptic currents; mIPSCs, miniature inhibitory postsynaptic currents.

After ten days of adaptive feeding (the Sham and CCH groups were housed in the standard environment, while the CCH+EE group was housed in the enriched environment), rats underwent 2-VO surgery. Following surgery, cerebral blood flow (CBF) measurements were taken. After 40 days, CBF was measured again, and the Morris water maze (MWM), novel object recognition (NOR), electrophysiological recording, western blot (WB), enzyme-linked immunosorbent assay (ELISA), and immunofluorescence (IF) tests were done. Figure 1 shows the protocols for the overall experiments.

### 2.3. Permanent bilateral common carotid artery occlusion

Rats were not fed for a whole day and anesthetized with 1% pelltobarbitalum natricum (P3761, SIGMA) 40 mg/kg intraperitoneal (i.p.). Through a midline cervical incision, rats were exposed to the unilateral common carotid arteries, which were then ligated using 5-0 silk sutures. Sham group rats also received the same treatment without being ligated the common carotid arteries. To reduce acute cerebral ischemic injury and optic nerve damage caused by acute vascular occlusion, we ligated the other lateral common carotid artery a week later (Wang et al., 2019).

### 2.4. Enriched environment intervention

The EE protocol was followed exactly as described (Alvarez et al., 2016). It was made up of a custom-made cage (90 cm × 90 cm × 45 cm) with a wire-mesh roof, a stainless-steel bottom, and glass walls. The roof can be removed to allow the rats access. The cage contained a variety of toys, including tunnels, hammocks, stairs, running wheels, game paper strips, platforms, and a seesaw made of wood or metal. These items were moved 3 times and cleaned once per week. In each cage, 10 rats were housed together and had free access to water and food.

### 2.5. Cerebral blood flow measurement

CBF was measured with a needle probe and a Brain Laser Doppler (BLD) device (Jarfalla, PeriFlux System5000, Perimed, Sweden) (Reference No. 411, Perimed). Via double-sided tape, the probe was attached to the stereotactic frame with a monitor. Rats were put in the stereotaxic frame. An electric drill was then used to drill a burr hole (d: 2 mm) through a midline scalp incision in the temporoparietal region (posterior: 4.8 mm; lateral: 2.5 mm). In this manner, a placement device for the contact probe was established. The probe collected the backscattered light, and the light wavelength change is proportional to the velocity of red blood cells in the research area, resulting in a noninvasive CBF measurement presented as perfusion units. During this time, PeriSoft software 2.5.5 (Jarfalla, Perimed, Sweden) was constantly recording the data for later offline analysis.

### 2.6. Morris water maze test

**FIGURE 2:**
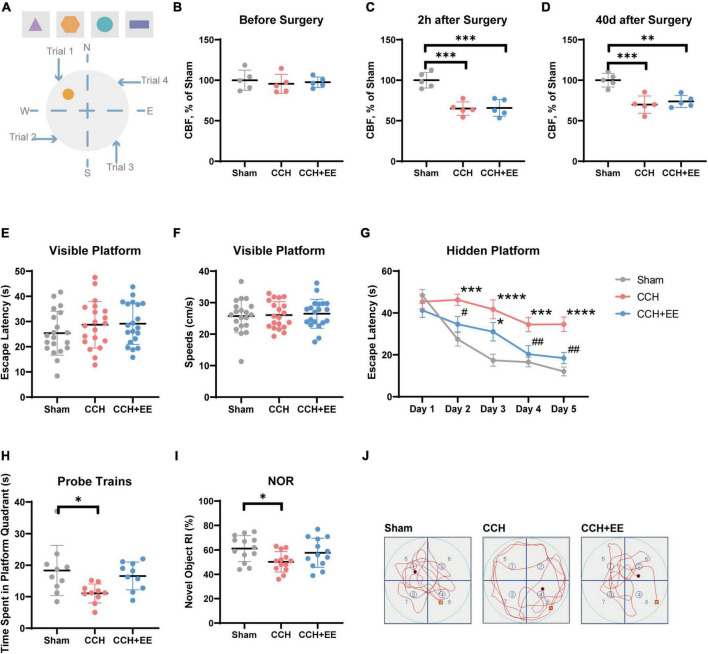
Description of behavioral test and CBF measurements. **(A)** Equipment setup of the maze, the orange circle indicates the location of the platform. Results of CBF measurements **(B)** before surgery, **(C)** 2 h after surgery and **(D)** 40 days after surgery in each group (*n* = 5 per group). Values are expressed as a percentage of the Sham group. **(E)** Escape latency and **(F)** speeds on the visible platform test (*n* = 20 per group). **(G)** Escape latency in hidden platform test. **(H)** Time spent in the target quadrant in the probe trains (*n* = 10 per group). **(I)** RI of novel object in NOR test (*n* = 10 per group). **(J)** Representative swimming paths of the Sham, CCH, CCH+EE groups. The data are presented as the mean ± SD; *p < 0.05, ***p < 0.001, ****p < 0.0001 compared to Sham group; ^#^*p* < 0.05, ^##^*p* < 0.01 compared to CCH group. NOR, novel object recognition; RI, recognition index.

Morris water maze was used to assess spatial learning and reference memory in rodents, and it was divided into three stages: the visible platform test, the hidden platform test, and probe trains. The MWM equipment was in the shape of a round pool (d:150 cm, h: 50 cm) and was filled with non-toxic dark water that was 32 cm deep and 24 ± 1°C. Meanwhile, the pool wall had been outfitted with a variety of orientation cues (Figure 2A). The pool was then divided into four equal quadrants.

During the visible platform test, a platform was exposed 1 cm above the water. A red flag was placed on the platform. The rat was dropped into the pool from the third quadrant. The time it took rats to reach the platform within 1 min (escape latency) was recorded using the Animal Video Tracking Analysis System (Anilab Scientific Instruments, Ningbo, China).

During the hidden platform test, the platform was about 2 cm below the water. In each trail, the rat was encouraged to find the platform for a maximum of 1 min. If it does not reach the platform within the time limit, the tester must guide the rat to the platform. The rat would be allowed to rest for 15 s once it arrived at its destination. The training phase will last 5 days, with four trails per day. On day 6, the platform was removed, and the rat was placed in the third quadrant and chased for 1 min. The swimming time of the rat in the quadrant was then recorded. After the trail, gently dry the rat and place it back in the cage.

### 2.7. Novel object recognition test

Rats were placed in the training room for 24 h prior to training to allow them to adjust to their surroundings. An open recognition box (60 cm × 60 cm × 40 cm) with a black underside and white walls served as the experimental setup. On training day, two identical objects (A and B) were placed at opposite ends of a single wall. The rat was allowed to freely explore both objects for 10 min. On test day, the old object B was replaced by an entirely new object C. For 10 min, rats were subjected to the box. Investigators recorded the time the rat spent exploring the objects. Recognition index (RI) was calculated as the formula:


$$RI=\frac{\text{time off novel object}}{\text{time of novel object + time of familiar object}}\times 100\%$$

### 2.8. Placement of electrodes

Rats were anesthetized and placed in a stereotactic frame. After exposing the skull, drill at the anteroposterior (4.8 mm) and mediolateral (2.5 mm) coordinates from the bregma. The recording electrode was placed in the dorsal CA1 pyramidal cell layer (dorsoventral: 3.6 mm). Each electrode was made from 2 cm stainless steel pieces with a 0.127 mm diameter. The reference electrode was placed 4 mm from the recording electrode on the skull. Dental cement (YY0270.1, DaJin, China) was used to secure the electrodes.

### 2.9. Local field potential recording and analysis

The local field potentials (LFPs) of dCA1 were recorded 3 days after electrode implantation. The ground electrode was clamped to the ear rim skin of the rat. The LFP signal was amplified by an external preamplifier (Octal Bio Amp, ADINSTRUMENTS, Australia), then filtered (1–100 Hz), and recorded using LabChart8b software (PowerLab 16/35, Australia). The spontaneous LFP was recorded after the LFP waveform became stable.

The LFPs analysis was carried out in MATLAB R2021a (MathWorks, USA) using the “Brainstorm” package. We used a Nortch filter to remove 50 Hz AC interference and a Fast Fourier Transform (FFT) to calculate the power spectrums. Morlet wavelets were used to compute the energy spectrums. Modulation index (MI) was used to calculate phase-amplitude coupling (PAC).

### 2.10. Western blot

To extract rat hippocampus protein, RIPA lysis buffer (P0013B; Beyotime Biotechnology) containing Pierce™ Protease Inhibitor Tablets (A32961; Thermo Scientific™) was used. The extract was then centrifuged to collect the supernatant. For each sample, 40 g protein was loaded onto SDS–PAGE gels (PG111, EpiZyme Biotech) and then transferred to polyvinylidene fluoride membranes (ISEQ00010, 0.22 mm, Millipore). The membranes were blocked for 2 h in Protein-free Rapid Blocking Buffer (PS108p, EpiZyme Biotech), and then incubated overnight at 4°C with diluted primary antibodies, including anti-VAChT (1:1,000, 139103, Synaptic Systems), anti-VGAT (1:1,000, 131003, Synaptic Systems), anti-VGLUT1 (1:1,000, 135011, Synaptic Systems) and anti-β-actin (1:5,000, ab6276, Abcam). Secondary antibodies, including goat anti-mouse (1:10,000, AS1106, ASPEN) and goat anti-rabbit (1:10,000, AS1107, ASPEN), were used for membrane application for 2 h at room temperature. The ImageJ software (NIH, USA) was used to calculate the semi-quantitation and visualization of targeted protein expression using Tanon-5200 (Tanon, China).

### 2.11 Enzyme-linked immunosorbent assay

To assess hippocampus glutamate (Glu), gamma-aminobutyric acid (GABA), and acetylcholine (Ach) levels, we used the Rat Glutamic Acid ELISA Kit (JYM0952Ra, JYMBIO), Rat Gamma-aminobutyric Acid ELISA Kit (JYM0325Ra, JYMBIO), and Rat Acetylcholine ELISA Kit (ml003048, Shanghai Enzyme-linked Biotechnology Co., Ltd.). We followed all the manufacturer’s instructions for the procedure. For Glu and GABA, we expressed the results in ng/mg protein, and for Ach, we expressed the results in μg/mg protein.

### 2.12. Immunofluorescence

For pre-fixing and post-fixing, 4% paraformaldehyde (#71061350, Biosharp) was used. A vibratome was used to cut fifty-micrometer sections (3 sections per rat) (VT1000s, Leica). The sections were then incubated for 1 h in a solution containing 10% goat serum (abs933, Absin) in saline buffered with phosphate and 0.25% Triton X-100 (abs47048168, Absin). Finally, the primary antibodies were incubated for 1 day at 4°C with the following antibodies: rabbit anti-VAChT (1:2,000, 139103, Synaptic Systems); rabbit anti-VGAT (1:1,000, 131003, Synaptic Systems); and rabbit anti-NeuN (1:1,000, ab177487, Abcam). Sections were rinsed in tris buffered saline for 10 min before being incubated for 12 h at 4°C with Alexa 488 or Alexa 594 (1:2,000, #4412/#8889, Cell Signaling Technology). The sections were rinsed three times in TBS for 10 min before mounting with the ProLong^®^ Gold Antifade Reagent with DAPI (#8961, Cell Signaling Technology). Following that, the sections were examined using a microscope with confocal laser scanning (TCS SP8, Leica, Germany), with four images taken from each section, including CA1, CA2, CA3, and DG parts. The quantification of staining occurred using the mean gray value *via* ImageJ software (NIH, Bethesda, MD, United States).

### 2.13. Whole-cell patch clamp recording

CA1-containing coronal brain slices (300 μm) were produced. Slices were placed in a recovery chamber with artificial cerebrospinal fluid (ACSF) with the following concentrations (in mM): NaCl 150.0, KCl 2.0, MgCl2 1.0, CaCl2 2.0, Hepes 10.0, and D-glucose 10.0 bubbled with a mixture of 5% CO_2_ and 95% O_2_ for 1 h at standard room temperature. Pyramidal neurons were subjected to whole-cell voltage clamp recordings using a HEKA/EPC-10 amplifier (HEKA, Lambrecht, Germany). The patch recording pipettes (5–7 MΩ) were filled with a solution containing (in mM): KCl 145.0, NaCl 5.0, Hepes 10.0, EGTA 5.0, and Na_2_ATP 4.0. Under a voltage clamp at-70 mV, miniature excitatory postsynaptic currents (mEPSCs) were recorded in the presence of tetrodotoxin (1 μM), D-AP5 (50 μM), and picrotoxin (50 μM). The picrotoxin in ACSF was substituted with CNQX (20 μM) for recordings of miniature inhibitory postsynaptic currents (mIPSCs). Clampfit 10.6 (Molecular Devices, Sunnyvale, CA, USA) software was then used to examine the frequencies and amplitudes of mEPSCs and mIPSCs.

### 2.14. Statistical analysis

IBM^®^ SPSS^®^ Statistics software v. 24 (IBM Corp., Armonk, NY, USA) was used to perform statistical analysis. Prior to data analysis, the Shapiro–Wilk test was used to assess data normality, and a variance homogeneity (Levene test) test was also run. The data in the escape latency during the space navigation training stage was analyzed using the two-way repeated ANOVA (analysis of variance) and the Bonferroni’s test *post-hoc*. Others were carried out using the one-way ANOVA with Tukey’s *post-hoc*. Before quantification, all results were scored while blinded. Correlation analysis was performed in GraphPad Prism 8.3.0 (GraphPad Software, Inc.). Mediation analysis was performed in R-Studio (JJ Allaire, United States) using the “bruceR” package. The value of *p* < 0.05 was considered statistically significant.

## 3. Results

### 3.1. The modified 2-VO surgery reduces cerebral blood flow while maintaining motor capacity

To determine whether the modified 2-VO surgery was effective, we used a laser doppler device to measure hippocampal CBF in the three groups. The results are shown in Figures 2B–D. CBF was similar in all three groups before surgery [*F* (2, 12) = 0.2276, *p* = 0.7998; Figure 2B], and significantly different 2 h later [*F* (2, 12) = 22.06, *p* < 0.0001; Figure 2C], and 40 days later [*F* (2, 12) = 16.90, *p* = 0.0003; Figure 2D]. More specifically, the surgery significantly reduced hippocampus CBF in the CCH group (*p* = 0.0002) and the CCH+EE group (*p* = 0.0003) and lasted for 40 days (CCH, *p* = 0.0005; CCH+EE, *p* = 0.0015). Additionally, our results showed that the CBF in the CCH+EE group had no significant differences compared with the CCH group 40 days post-operation (*p* = 0.7701). According to the above findings, the modified 2-VO surgical procedure successfully reduced CBF.

The visible platform test was performed to detect the effect of the modified 2-VO on the visual and sensorimotor abilities in rats (Popelikova et al., 2018). There was no significant difference in swimming speed between the three groups [*F* (2, 57) = 0.02281, *p* = 0.9775; Figure 2F], indicating that there was no damage to sensorimotor ability. Similarly, the escape latency was not significantly different between the three groups [*F* (2, 57) = 0.07728, *p* = 0.9257; Figure 2E], which shows that the effect of the modified 2-VO surgery on vision will not reduce the reliability of behavioral test results.

### 3.2. Enriched environment improved the spatial learning and memory abilities in chronic cerebral hypoperfusion rats

Rats were trained for five consecutive days to assess their spatial learning ability in the hidden platform test. The two-way repeated-measures ANOVA indicated that the escape latency decreased with the increase of training days in all three groups [*F* (4, 135) = 24.26, *p* < 0.0001], with a significant difference between groups [*F* (2, 135) = 30.72, *p* = 0.0001; Figure 2G]. From day 2 to day 5, the escape latency of the CCH group was significantly higher than that of the Sham group (*p* < 0.01). The escape latency of the CCH+EE group was significantly shorter than the CCH group on day 2 (*p* = 0.0439), day 4 (*p* = 0.0099), and day 5 (*p* = 0.0025). Furthermore, with the exception of day 3 (*p* = 0.0137), there was no significant difference between the Sham group and the CCH+ EE group.

In probe trains, the difference in time spent in the platform quadrant between the three groups was significant [*F* (2, 27) = 4.693, *p* = 0.0178; Figure 2H]. The CCH group spent considerably less time in the platform quadrant than the Sham group (*p* = 0.0178). There was no significant difference between the CCH+EE and Sham groups (*p* = 0.7586). Figure 2J shows the representative swimming trajectories of the rats in probe trains. It can be clearly observed that the trajectories of the Sham and CCH+EE groups were more complex in the platform (upper left) quadrant, while the swimming trajectories of the rats in the CCH group were more average in all quadrants.

In the NOR test, the performances of the three groups differed significantly [*F* (2, 36) = 3.724, *p* = 0.0339; Figure 2I]. The novel object recognition index of the CCH group was significantly lower than that of the Sham group (*p* = 0.0294). There was no significant difference between the CCH+EE group and the Sham group (*p* = 0.6647). Overall, EE improved the spatial learning and memory abilities of CCH rats.

### 3.3. Enriched environment modified theta and gamma rhythm power distributions in chronic cerebral hypoperfusion rats

**FIGURE 3:**
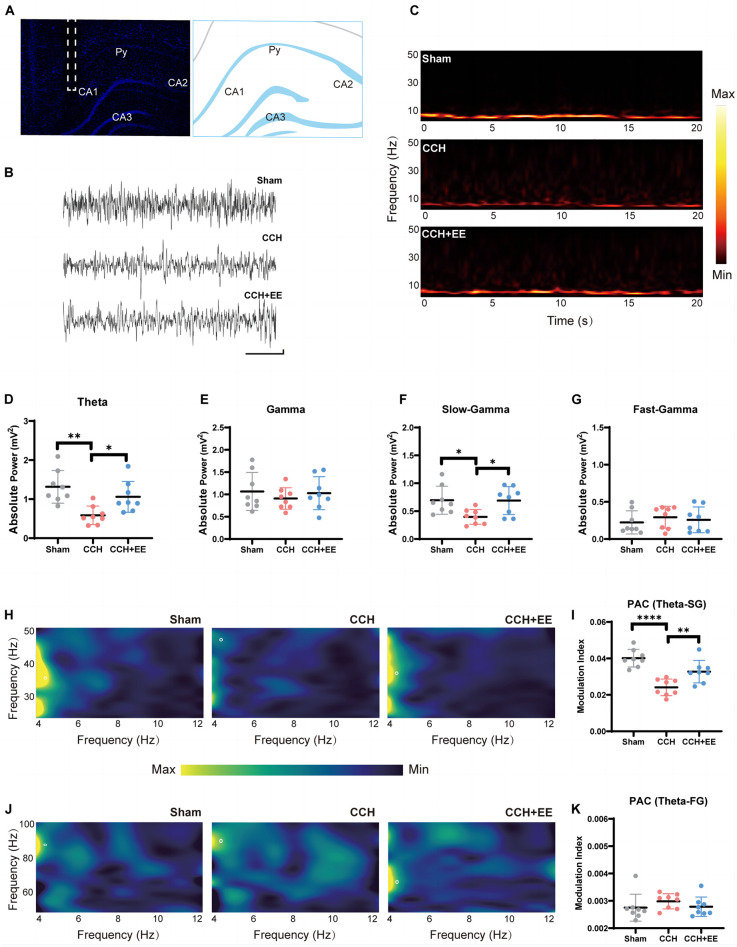
Local field potential recording and power analysis in three groups. **(A)** Schematic depiction of the recording (CA1) electrode placement in the *in vivo* electrophysiological experiment. **(B)** Example of the original signal of the field potential from the CA1 region. Scale bars = 1 s/0.1 mV. **(C)** CA1 time-frequency within 20 s recordings. The color bar was displayed in the graphics’ normalized scales. The more vibrant the hue, the greater the power. Comparison of **(D)** the theta power, **(E)** gamma power, **(F)** slow-gamma power and **(G)** the fast-gamma power. **(H)** Typical PAC between CA1 theta phase and SG amplitude in three groups. **(I)** Comparison of the modulation index of theta rhythm and SG rhythm. **(J)** Typical PAC between CA1 theta phase and FG amplitude in three groups. **(K)** Comparison of the modulation index of theta rhythm and FG rhythm (*n* = 8 per group). The data are presented as the mean ± SD; *p < 0.05, **p < 0.01, ****p < 0.0001. PAC, phase amplitude coupling; SG, slow gamma; FG, fast gamma.

We recorded LFPs from the hippocampal CA1 area (Figures 3A, B). Our results show a significant difference in theta power [*F* (2, 21) = 8.487, *p* = 0.0020, Figures 3C, D], but no significant difference in gamma power [*F* (2, 21) = 0.4334, *p* = 0.6539, Figure 3E]. Concretely, the 2-VO resulted in a decrease in theta power (*p* = 0.0016) but no change in gamma power (*p* = 0.6508). The EE intervention restored the power of theta (*p* = 0.0398). Multiple studies have recently agreed that rhythmic activity over the vast frequency range designated as gamma really contains more than one form of brain rhythm (Colgin, 2016). We further divided the gamma rhythm into two sub-bands: fast gamma (FG, 50–100 Hz) rhythm and slow gamma (SG, 30–50 Hz) rhythm. We discovered significant differences in the SG [*F* (2, 21) = 4.844, *p* = 0.0186, Figure 3F] and no differences in the FG [*F* (2, 21) = 0.3859, *p* = 0.6846, Figure 3G]. The CCH group had lower SG rhythm power (*p* = 0.0326) than the Sham group, and EE intervention increased the low power in the CCH rat (*p* = 0.0370).

The above indicated that the power distributions of theta and SG rhythms in the hippocampus neural network were seriously disturbed in CCH rats, and EE altered the abnormal power distribution.

### 3.4. Enriched environment enhanced the phase-amplitude coupling of theta-SG in chronic cerebral hypoperfusion rats

We investigated the neural communication between theta and gamma rhythms in the three groups. We computed stable, power-controlled coupling distributions per the modulation index (MI) method to assess the phase-amplitude coupling (PAC). First, we analyzed the MI of theta and SG rhythms. The one-way ANOVA showed significance [*F* (2, 21) = 18.71, *p* < 0.0001, Figures 3H, I] in all three groups. MI of theta-SG rhythm in CCH group was significantly decreased compared with the Sham group (*p* < 0.0001), and EE increased MI in CCH rats (*p* = 0.0094). We next calculated the MI of theta-FG. One-way ANOVA showed no significant difference in all three groups [*F* (2, 21) = 1.942, *p* = 0.1683, Figures 3J, K]. These findings suggest that 2-VO reduces theta-SG coupling and that EE improves the reduction of the coupling caused by CCH.

### 3.5 Enriched environment restores neurotransmitter levels and influences electrical activity *via* presynaptic afferent pathways

**FIGURE 4:**
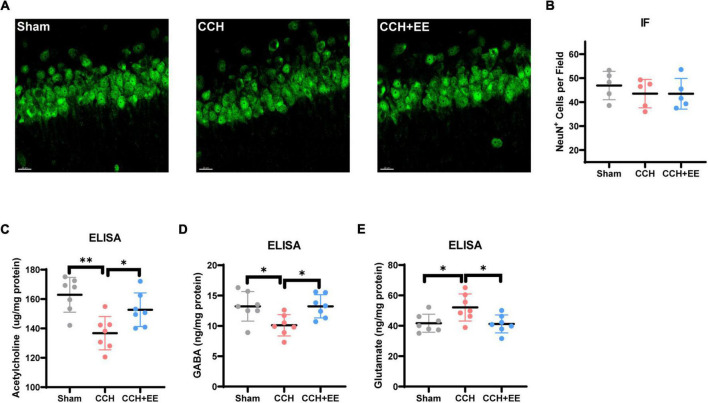
NeuN immunostaining results and ELISA results for three neurotransmitters. **(A)** IF staining for NeuN in CA1. Scale bars = 20 μm. **(B)** Comparison of NeuN quantification by IF (*n* = 5 per group). The ELISA results of **(C)** acetylcholine, **(D)** GABA and **(E)** glutamate (*n* = 7 per group). The data are presented as the mean ± SD; *p < 0.05, **p < 0.01.

To investigate the causes of the alterations in neural oscillations, we further stained neurons in the rat hippocampus. The NeuN immunofluorescence results revealed that there was no discernible change in the number of neurons in the CA1 across the three groups [*F* (2, 21) = 0.5247, *p* = 0.6047, Figures 4A, B]. This indicates that there may be a more fundamental explanation for the variations in the electrical activity of neural clusters. By using ELISA tests, we discovered that in CCH rats, the homeostasis of three classical neurotransmitters was disrupted, whereas EE restored the disturbed neurotransmitter levels. More specifically, the hippocampal Ach (*p* = 0.0014) and γ-aminobutyric acid (GABA) (*p* = 0.0271) levels were decreased and glutamate (*p* = 0.0337) was increased in CCH rats (Figures 4C–E). EE increased Ach (*p* = 0.0470), GABA (*p* = 0.0271), and decreased glutamate (*p* = 0.0262) in CCH rats.

**FIGURE 5:**
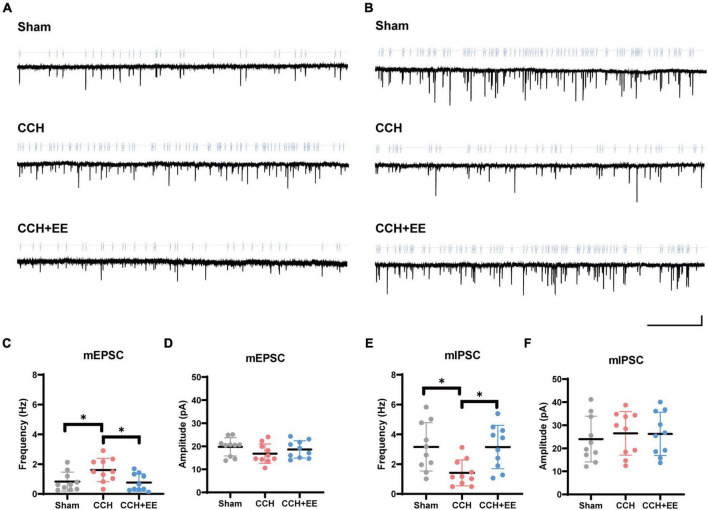
The results of whole-cell patch clamp recording. Traces of **(A)** mEPSCs and **(B)** mIPSCs obtained in the CA1 pyramidal neuron. Scale bars = 5 s/15 pA. **(C)** mEPSCs frequency and **(D)** amplitude from 3 groups. **(E)** mIPSCs frequency and **(F)** amplitude from 3 groups. The data are presented as the mean ± SD; *p < 0.05.

We next performed the whole-cell patch clamp recording. We measured mEPSCs and mIPSCs to investigate the synaptic characteristics of pyramidal neurons (Figures 5A, B). In the CCH group, the frequency of mEPSCs was enhanced (*p* = 0.0409) without a significant change in amplitude (*p* = 0.2433). In the CCH+EE group, pyramidal neurons showed similar mEPSC frequencies (*p* = 0.9743) and amplitudes (*p* = 0.7982) to those in the Sham group (Figures 5C, D). For mIPSCs, the frequency was lower in the CCH group compared to the other two groups (*p* = 0.0208, *p* = 0.0216), but the amplitude was not significantly different [*F* (2, 27) = 0.2047, *p* = 0.8161, Figures 5E, F]. Generally, changes in the amplitude of spontaneous events are thought to be caused by postsynaptic processes, whereas changes in spontaneous event frequency are thought to be generated by presynaptic mechanisms. Our results indicate that presynaptic afferent pathways may play a significant role in the alteration of electrical activity caused by CCH and EE.

### 3.6. Enriched environment restored the afferent balance in the chronic cerebral hypoperfusion rats’ hippocampus

**FIGURE 6:**
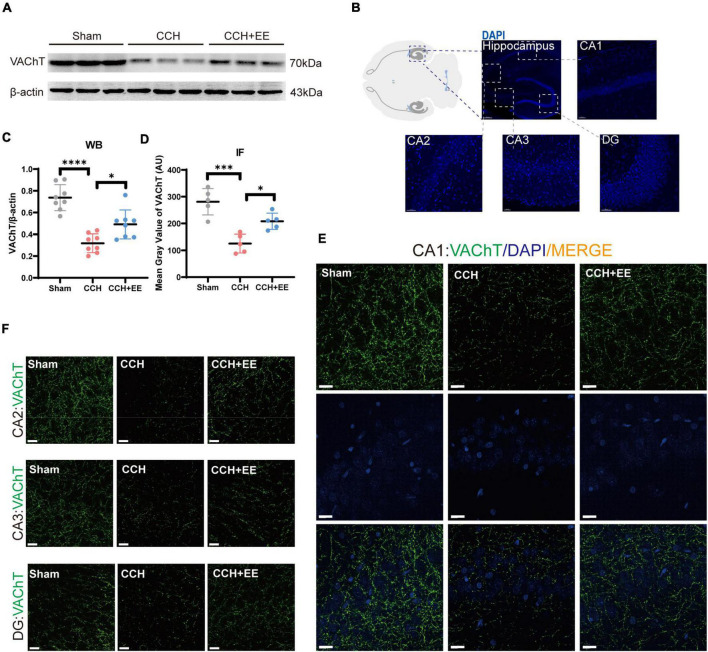
Altered VAChT in hippocampus of three groups. **(A)** WB of tissue samples from hippocampus, immunolabeled for VAChT and β-actin. **(B)** Schematic representation of the IF position. **(C)** Relative quantification of VAChT. β-actin was used as a loading control (*n* = 8 per group). **(D)** Comparison of VAChT quantification by IF (*n* = 5 per group). **(E)** IF staining for VAChT in CA1. Scale bars = 20 μm. **(F)** IF staining for VAChT in CA2, CA3 and DG. Scale bars = 20 μm. The data are presented as the mean ± SD; *p < 0.05, ***p < 0.001, ****p < 0.0001. VAChT, vesicular acetylcholine transporters.

**FIGURE 7:**
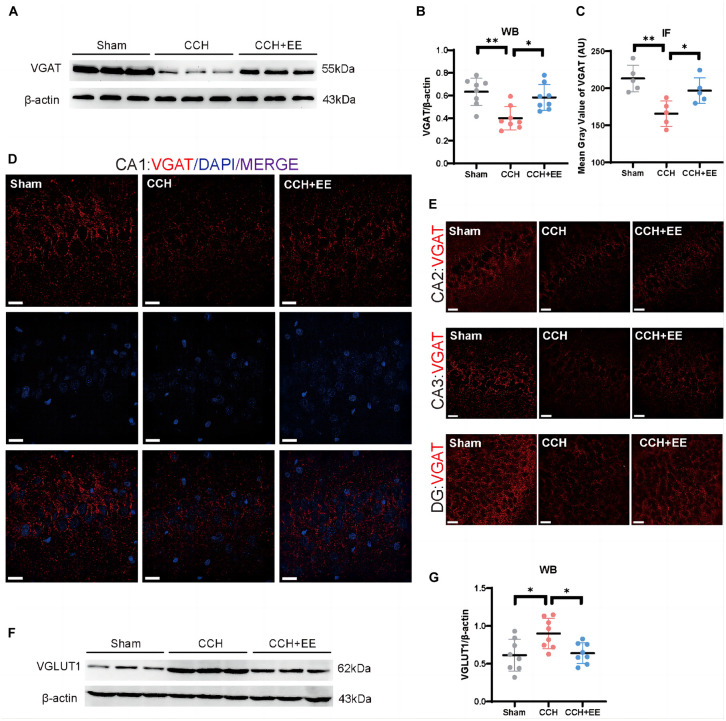
Altered VGAT and VGLUT1 in hippocampus of three groups. **(A)** WB of tissue samples from hippocampus, immunolabeled for VGAT and β-actin. **(B)** Relative quantification of VGAT. β-actin was used as a loading control (*n* = 8 per group). **(C)** Comparison of VAChT quantification by IF (*n* = 5 per group). **(D)** IF staining for VGAT in CA1. Scale bars = 20 μm. **(E)** IF staining for VGAT in CA2, CA3 and DG. Scale bars = 20 μm. **(F)** WB of tissue samples from hippocampus, immunolabeled for VGLUT1 and β-actin. **(G)** Relative quantification of VGLUT1. β-actin was used as a loading control (*n* = 8 per group). The data are presented as the mean ± SD; *p < 0.05, **p < 0.01. VGAT, vesicular GABA transporters; VGLUT1, vesicular glutamate transporter 1.

To define the three presynaptic innervations of the hippocampus, we used vesicular transporters (VTs) of three classical neurotransmitters situated in the synaptic vesicle membrane. Vesicular acetylcholine transporters (VAChT), vesicular GABA transporters (VGAT), and vesicular glutamate transporter 1 (VGLUT1) were used to label the cholinergic, GABAergic, and glutamatergic neural inputs to the hippocampus (Helmuth, 2000; Fremeau et al., 2004; Riazanski et al., 2011; Al-Onaizi et al., 2017). Figure 6B shows the locations of the designated hippocampal subdivisions during the immunofluorescence quantification performed in this study. WB and IF revealed a significant difference in VAChT between the three groups [WB: *F* (2, 21) = 27.07, *p* < 0.0001; Figures 6A, C; IF: *F* (2, 12) = 19.81, *p* = 0.0002; Figures 6D–F]. The expression of VAChT in the CCH group was significantly lower than in the Sham group (WB: *p* < 0.0001; IF: *p* < 0.001). EE increased VAChT levels in CCH rats (WB: *p* = 0.0170; IF: *p* = 0.0151). Similarly, both WB and IF revealed a significant difference in VGAT expression between the three groups [WB: *F* (2, 21) = 9.506, p = 0.0011; Figures 7A, B; IF: *F* (2, 12) = 9.491, *p* = 0.0034; Figures 7C–E]. The expression of VGAT in the CCH group was significantly lower than in the Sham group (WB: *p* = 0.0013; IF: *p* = 0.0028), and EE increased VGAT expression in CCH rats (WB: *p* = 0.0100; IF: *p* = 0.0394). For glutamatergic neural inputs, the level of VGLUT1 [*F* (2, 21) = 5.832, *p* = 0.0097; Figures 7F, G] was significantly elevated in the CCH group (*p* = 0.0148). EE reduced the elevated VGLUT1 in CCH rats (*p* = 0.0272). These findings indicate that the homeostasis of the hippocampal afferent nerves is disrupted in CCH rats and that EE can restore this balance.

### 3.7. Association analysis and mediation analysis

Given the results above, we first assessed the association of theta power, SG power, and the strength of the PAC of theta-SG with cognitive level (Table 1). We discovered that improved spatial learning and memory capacities were related to increased theta rhythm power (*p* = 0.025) and a stronger PAC of theta-SG (*p* = 0.002). No indicators were associated with recognition memory (Table 1). Then, we evaluated the relationship between the three VTs and cognitive levels (Table 2). Better behavioral performance in the MWM was associated with elevated VAChT (*p* = 0.024) and VGAT (*p* = 0.041) and was not associated with VGLUT1 (*p* = 0.091). For NOR experiments, the expression levels of all three proteins did not correlate with the RI (Table 2). Last, we investigated the correlation of the three VTs with theta power, SG power, and the strength of the PAC of theta-SG (Table 3). Theta power was positively correlated with the expression levels of VAChT (*p* < 0.001) and VGAT (*p* = 0.004) but not with the levels of VGLUT1 (*p* = 0.407). The elevated power of SG was associated with an increase in VGAT (*p* = 0.026) and was not correlated with the remaining two proteins (VAChT: *p* = 0.077; VGAT: *p* = 0.506). All three VTs were shown to be linked with PAC, where VAChT (*p* = 0.001) and VGAT (*p* = 0.008) were positively correlated, and VGLUT1 (*p* = 0.037) was negatively correlated.

**TABLE 1:** Association analysis between neural oscillations and cognitive functions.

Marker	WMW		NOR	
	**RS (95% CI)**	***p***	**RS (95% CI)**	***p***
Theta	0.4566 (0.05264 to 0.7322)	0.025*	0.1132 (−0.3155 to 0.5035)	0.598
SG	0.0300 (−0.3888 to 0.4385)	0.889	−0.0601 (−0.4625 to 0.3629)	0.780
PAC (Theta-SG)	0.6107 (0.2633 to 0.8179)	0.002*	0.2049 (−0.2284 to 0.5704)	0.337

RS, spearman coefficient of rank correlation; WMW, experimental results of probe trains in the morris water maze; NOR, recognition index of novel object recognition; SG, slow-gamma; PAC, phase amplitude coupling. *p < 0.05.

**TABLE 2:** Association analysis between vesicular transporters and cognitive functions.

Marker	WMW		NOR	
	**RS (95% CI)**	***p***	**RS (95% CI)**	***p***
VAChT	0.4587 (0.0552 to 0.7334)	0.024*	0.2316 (−0.2017 to 0.5891)	0.276
VGAT	0.4205 (0.0080 to 0.7107)	0.041*	−0.0059 (−0.4188 to 0.4090)	0.978
VGLUT1	−0.3527 (−0.6690 to 0.0717)	0.091	−0.1559 (−0.5353 to 0.2759)	0.467

RS, spearman coefficient of rank correlation; WMW, experimental results of probe trains in the morris water maze; NOR, recognition index of novel object recognition; VAChT, vesicular acetylcholine transporters; VGAT, vesicular GABA transporters; VGLUT1, vesicular glutamate transporter 1. *p < 0.05.

**TABLE 3:** Association analysis between vesicular transporters and neural oscillations.

Marker	Theta		SG		PAC (Theta-SG)	
	**RS (95% CI)**	***p***	**RS (95% CI)**	***p***	**RS (95% CI)**	***p***
VAChT	0.6658 (0.3477 to 0.8465)	<0.001*	0.3678 (−0.0543 to 0.6785)	0.077	0.6314 (0.4116 to 0.8663)	0.001*
VGAT	0.5626 (0.1282 to 0.7656)	0.004*	0.4544 (0.0499 to 0.7309)	0.026*	0.5289 (0.2737 to 0.8215)	0.008*
VGLUT1	−0.1774 (−0.5534 to 0.2519)	0.407	0.1426 (−0.2883 to 0.5255)	0.506	−0.4275 (−0.7283 to −0.0445)	0.037*

RS, spearman coefficient of rank correlation; SG, slow-gamma; PAC, phase amplitude coupling; VAChT, vesicular acetylcholine transporters; VGAT, vesicular GABA transporters; VGLUT1, vesicular glutamate transporter 1. *p < 0.05.

**FIGURE 8:**
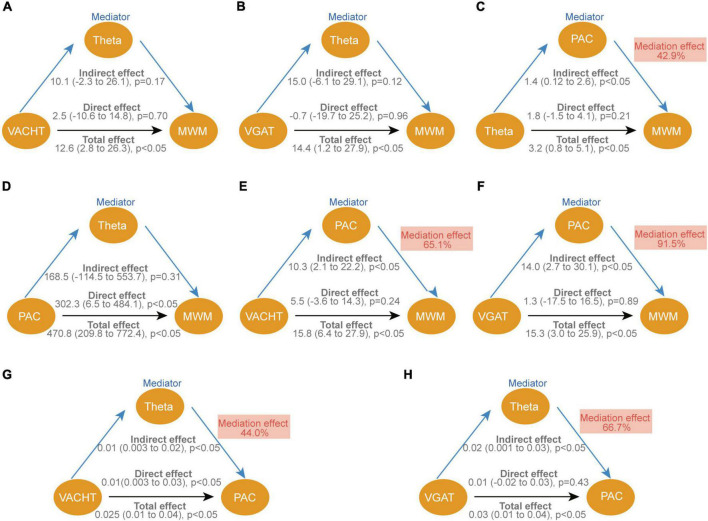
Causal mediation analysis for VAChT, VGAT, theta and PAC of theta-SG on cognitive function. **(A–H)** The standardized regression coefficients correcting for variables associated with each path in the model are depicted in diagrams. Each graphic shows the bootstrap statistical significance (*p*-values) of the direct and indirect approaches. The indirect impact is reported as a percentage of the total effect as the mediation effect. VAChT, vesicular acetylcholine transporters; VGAT, vesicular GABA transporters; PAC, phase amplitude coupling of theta-SG; MWM, experimental results of probe trains in the morris water maze.

Significantly correlated results were selected in the above association analysis for mediation analysis. The mediation analysis outlines three main assumptions. First, the direct effects of VAChT (*p* = 0.702), VGAT (*p* = 0.956), and theta rhythm (*p* = 0.207) on behavioral performance in the MWM were not significant (Figures 8A–C), whereas the PAC of theta-SG (*p* = 0.011) directly affected the outcome of the MWM (Figure 8D). Second, both VAChT (*p* = 0.041) and VGAT (*p* = 0.046) can affect behavioral performance indirectly *via* PAC (Figures 8E, F). At the same time, we found that the effect of VAChT on PAC was partly direct (*p* = 0.029) and partly indirect through modulation of theta rhythm (*p* = 0.027; Figure 8G). In contrast, VGAT only had an indirect influence *via* the theta rhythm on PAC (*p* = 0.027; Figure 8H).

## 4. Discussion

**FIGURE 9:**
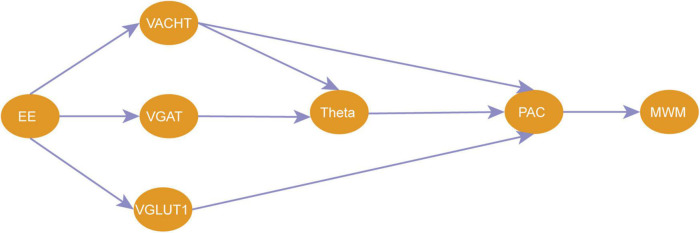
A pathway for EE to improve cognitive impairment due to CCH. EE, enriched environment; VAChT, vesicular acetylcholine transporters; VGAT, vesicular GABA transporters; VGLUT1, vesicular glutamate transporter 1; PAC, phase amplitude coupling of theta-SG; MWM, experimental results of probe trains in the morris water maze.

The results of this study indicate that CCH rats have altered hippocampal afferent neurotransmitters and neural oscillations, and that EE improves neural oscillations in the hippocampus by modulating the balance of hippocampal afferent nerves, thereby enhancing cognitive function. Specifically, EE altered the abnormal power distribution in CCH rats by increased the power in the theta and SG bands and the phase synchronization of theta-SG in CA1. This suggests that a significant improvement in spatial learning and memory deficits is closely associated with enhanced neural oscillation patterns. Additionally, higher theta power, SG power, and PAC were associated with a balance of cholinergic, GABAergic, and glutamatergic neurotransmitters in the hippocampus. And by association analysis and mediation analysis, we found that EE increased the power of theta rhythm by increasing the level of VAChT and VGAT, which then enhanced the PAC of theta-SG and finally led to an improvement in the cognitive level of CCH (Figure 9).

Chronic cerebral hypoperfusion is a common pathological state of the central nervous system (Raz et al., 2016). Narrow or occluded neck arteries cause CCH. A modified 2-VO has been established in this study to better understand the role of CCH in the development of cognitive dysfunction. The model was ligated twice one week apart to avoid acute cerebral ischemic injury and severe visual dysfunction caused by the sudden reduction of CBF (Wang et al., 2019). A visible platform experiment was used to test the locomotor ability and visual acuity of the rats after surgery, and the results showed that the speeds and escape latency of the CCH group were not significantly different from those of the Sham group, ensuring the dependability of subsequent behavioral experiments.

In the measurement of CBF, the experimental findings demonstrated that our 2-VO surgery significantly decreased CBF in rats, which stayed lower than the Sham group 40 days later, showing that the CCH model was successfully represented. In addition, the CBF of the CCH+EE group did not differ from that of the CCH group, indicating that EE did not work by restoring CBF. In the behavioral results, the modified 2-VO model exhibited cognitive dysfunction similar to the classical 2-VO model (Wang et al., 2019). The behavioral improvement of EE on CCH rats in this study reaffirms that EE is an effective intervention for CCH-related cognitive impairment.

Typically, the cognitive brain is formed by an interconnected network of neurons. The spatial organization of neuronal inputs is critical for neuronal network function (Colgin, 2016). In the hippocampus of CCH rats, our field potential analysis revealed an abnormal power distribution of CA1 (Figure 3). The power of theta and SG rhythms was significantly reduced in CCH rats, which is consistent with findings in other models of cognitive disorders (Gonzalez et al., 2022; Wang et al., 2022). EE improved the damaged theta and SG oscillations. According to the current literature, theta and SG plays critical roles in the higher functions of the brain. Theta rhythm is connected with a number of cognitively relevant actions, including spatial learning, memory, and sniffing movements. SG rhythm promotes memory recovery and is involved in memory retrieval (Colgin, 2016). In our study, rats with decreased theta and SG rhythms performed worse in the WMW and NOR test (Figures 2, 3), which is consistent with previous research (Kitanishi et al., 2015).

It has been observed that neural oscillations interact with one another *via* cross-frequency coupling. In cognitive-behavioral studies, it is common to see intermodulation of neural oscillations of different rhythms in the same brain region, such as PAC between theta and gamma rhythms. PAC means that the amplitude of the high rhythm is modulated by the phase of the low rhythm and is locked to the phase of the low rhythm. The PAC between theta and SG in the hippocampus of rodents and humans is mostly related to learning and memory (Karlsson et al., 2022). In our study, we found that cross-frequency coupling of theta-SG was impaired in CCH rats, indicating that information exchange between different frequency bands was impaired. EE improved the cross-frequency coupling along with the abnormal power distribution. What’s more, the results of our mediation analysis showed that the theta-SG PAC directly affected cognitive levels.

The possible mechanisms of PAC alteration are unclear (Lisman and Jensen, 2013). The results of our mediation analysis show that theta rhythm have the potential to modulate the PAC of theta and gamma. In recent years, a number of studies have presented evidence supporting this potential. For example, a study on fear states showed that there was a direct correlation between theta power and theta-gamma coupling and that theta enhanced coupling in a behaviorally relevant manner (Stujenske et al., 2014). In isolated hippocampal slices, light stimulation at theta frequency induces gamma oscillations nested in theta oscillations (Butler et al., 2016). A research in epileptic rats found that abnormal firing activity of interneurons affected their phase locking with respect to theta rhythm, eventually disrupting the coupling between theta and gamma in the hippocampus (Lopez-Pigozzi et al., 2016). The above evidence highlights the potential for modulation of PAC by theta rhythm, which is consistent with the results of our mediation analysis.

Now that there is a definite alteration in the neural oscillations in the CCH model, we sought to figure out the causes of the alteration. Firstly, we performed NeuN immunofluorescence staining, and we exclude the effect of neuronal death on neural oscillations. This indicates that there may be a more fundamental explanation for the variations in the electrical activity of neural clusters. Typically, neural oscillations in the hippocampus are modulated by afferent nerve fibers to the hippocampus *via* releasing various neurotransmitters. Several reports have described that CCH rats had cholinergic and GABAergic dysfunction as well as glutamate excitotoxicity (Qu et al., 2014; Long et al., 2015; Wang et al., 2020). Therefore, we performed ELISA experiments and confirmed that Ach and GABA levels decreased and glutamate levels increased in CCH rats. In the patch clamp experiment, the frequency but not the amplitude of spontaneous events changed significantly among the three groups. This suggests that presynaptic afferent pathways may also play an important role in the alteration of electrical activity caused by CCH and EE. In other words, presynaptic structures in the hippocampus associated with the release of the corresponding neurotransmitters may be key to disrupting the hippocampal network.

The hippocampus receives projections from various types of neurons from different brain regions. Vesicular transporters (VTs) situated on the synaptic vesicles (SVs) at these projecting neurons’ axon terminals, load neurotransmitters into the SVs and contribute to vesicle release (Vernon et al., 2019). Therefore, we examined the VTs levels of three classical neurotransmitters. As expected, CCH rats had lower VAChT, lower VGAT, and higher VGLUT1 levels and EE improved the disorder of these proteins, which is consistent with changes in the neurotransmitters corresponding to the three VTs. By association analysis and mediation analysis, we discovered that VAChT and VGAT can directly or indirectly influence theta to improve cognition. It has been shown GABAergic and cholinergic neurons projecting to the hippocampus from areas such as the medial septal nucleus generate and modulate theta rhythms in the CA1. The projecting GABAergic neurons operate as rhythmic oscillators, causing CA1 interneurons to produce phase-constant rhythmic theta oscillations. And cholinergic neurons can promote theta by inhibiting the generation of rhythms that are antagonistic to theta rhythms (Vandecasteele et al., 2014).

Enriched environment (EE) is a crucial experimental paradigm for understanding how interactions between genes and the environment alter the structure and function of the brain. It highlights motor, sensory, cognitive, and emotional or social stimuli as drivers, producing generalizing effects such as arousal or learning (Kempermann, 2019). How external conditions and behaviors influence changes in intrinsic biological molecules has always been a subject of intense interest and importance. In a study demonstrating that EE can alter gene expression levels through epigenetics, EE increased acetylation of chromatin bound to the ChAT gene promoter, thereby increasing ACh levels in the cholinergic circuit of stroke-affected mice (Wang et al., 2016). By increasing neurogenesis in the dentate gyrus, EE can increase GABA levels and activity-dependent GABA release in aged rats (Segovia et al., 2006). According to reports (Horvath et al., 2013), EE may reduce the excitatory effects of glutamate by promoting brain-derived neurotrophic factor. Additionally, EE can regulate glutamate levels by methylating DNA (Jeyaraj et al., 2021). Numerous hypotheses exist regarding the mechanism by which EE affects various neurotransmitter systems, but no consensus has yet been reached. In the present study, we describe the role of EE in regulating neurotransmitter homeostasis by modulating VTs of the respective neurotransmitter. However, we did not explore the mechanism further, which is a limitation of this paper and the next step of our future research.

## Conclusion

In conclusion, our study shows that hippocampal afferent neurotransmitters and neural oscillations are impaired in CCH rats and that EE improves neural oscillations in the hippocampus by modulating the balance of hippocampal afferent nerves. We propose a new pathway for EE to improve cognitive impairment due to CCH. These findings have important implications for the treatment of CCH-related cognitive impairment.

## Data availability statement

The raw data supporting the conclusions of this article will be made available by the authors, without undue reservation.

## Ethics statement

All the experiments were conducted pursuant to the Guide for the Care and Use of Laboratory Animals (Guide, NRC 2011) and the European Convention for the Protection of Vertebrate Animals Used for Experimental and Other Scientific Purposes (ETS 123) with the approval of the IACUC (IACUC Number: SQ20200054) of Wuhan University Center for Animal Experiment.

## Author contributions

JiZ, XL, and YL contributed to the study concept and design. JiZ, LC, TL, ZZ, and SP contributed to performing the animal study. YH, MZ, and LX collected and analyzed the data. JiZ drafted the manuscript. All authors commented on the manuscript, read, and approved the final manuscript.
