# Supplementary figures and images for: Enriched environment attenuates hippocampal theta and gamma rhythms dysfunction in chronic cerebral hypoperfusion via improving imbalanced neural afferent levels
Source: Front Cell Neurosci. 2023 May 17;17:985246. doi: 10.3389/fncel.2023.985246 (PMC10231328; doi:10.3389/fncel.2023.985246)

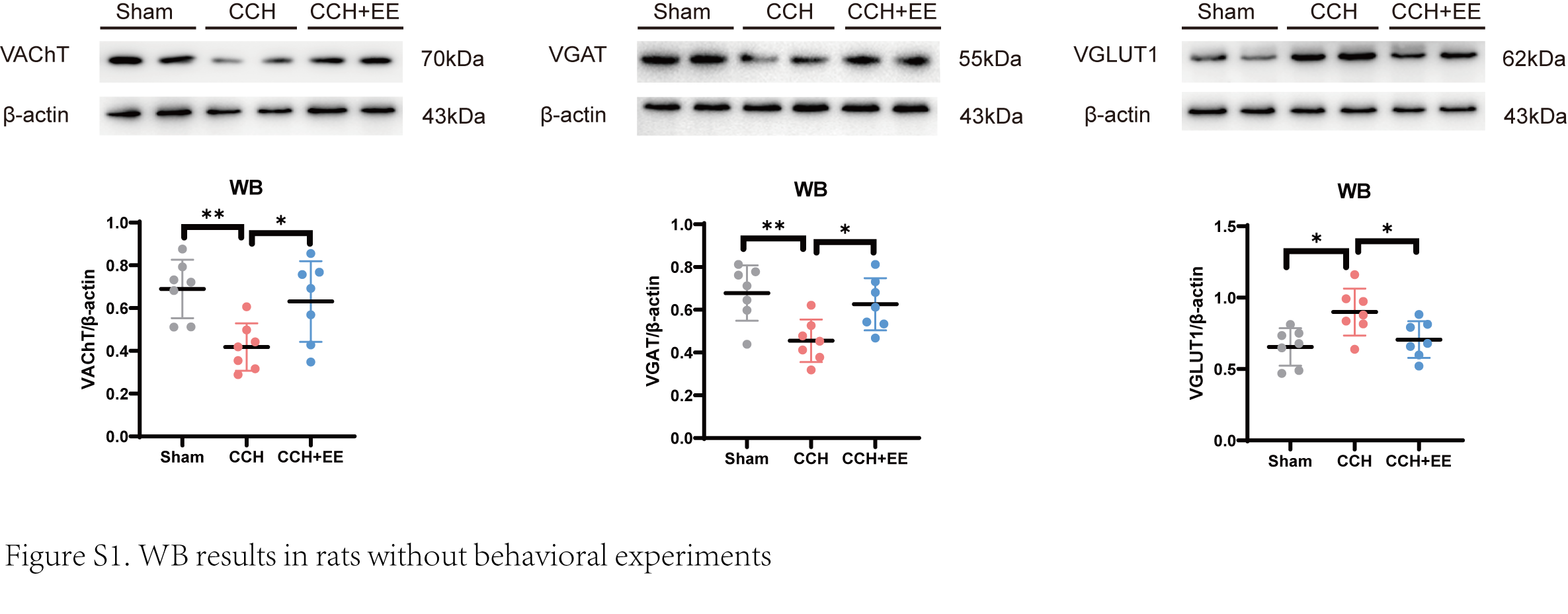

Supplement: Supplementary file 1 [file Image_1.TIF]
